# Screening the Toxicity of Selected Personal Care Products Using Embryo Bioassays: 4-MBC, Propylparaben and Triclocarban

**DOI:** 10.3390/ijms17101762

**Published:** 2016-10-21

**Authors:** Tiago Torres, Isabel Cunha, Rosário Martins, Miguel M. Santos

**Affiliations:** 1 CIMAR/CIIMAR, Laboratório Associado—Centro Interdisciplinar de Investigação Marinha e Ambiental, Universidade do Porto, Av. General Norton de Matos s/n, 4450-208 Porto, Portugal; torres_tapt@hotmail.com (T.T.); isabel.cunha@ciimar.up.pt (I.C.); mrm@estsp.ipp.pt (R.M.); 2 Escola Superior de Tecnologia de Saúde do Porto, Instituto Politécnico do Porto, 4200-072 Porto, Portugal; 3 FCUP-Departamento de Biologia, Faculdade de Ciências da Universidade do Porto, Rua do Campo Alegre, 4169-007 Porto, Portugal

**Keywords:** personal care products, sea urchin, zebrafish, embryo bioassays, risk assessment, 4-Methylbenzylidene Camphor, propylparaben, triclocarban, Registration, Evaluation, Authorization, and Restriction of Chemicals (REACH)

## Abstract

Recently, several emerging pollutants, including Personal Care Products (PCPs), have been detected in aquatic ecosystems, in the ng/L or µg/L range. Available toxicological data is limited, and, for certain PCPs, evidence indicates a potential risk for the environment. Hence, there is an urgent need to gather ecotoxicological data on PCPs as a proxy to improve risk assessment. Here, the toxicity of three different PCPs (4-Methylbenzylidene Camphor (4-MBC), propylparaben and triclocarban) was tested using embryo bioassays with *Danio rerio* (zebrafish) and *Paracentrotus lividus* (sea urchin). The No Observed Effect Concentration (NOEC) for triclocarban was 0.256 µg/L for sea urchin and 100 µg/L for zebrafish, whereas NOEC for 4-MBC was 0.32 µg/L for sea urchin and 50 µg/L for zebrafish. Both PCPs impacted embryo development at environmentally relevant concentrations. In comparison with triclocarban and 4-MBC, propylparaben was less toxic for both sea urchin (NOEC = 160 µg/L) and zebrafish (NOEC = 1000 µg/L). Overall, this study further demonstrates the sensitivity of embryo bioassays as a high-throughput approach for testing the toxicity of emerging pollutants.

## 1. Introduction

In the past few years, due to methodological and technological improvements, many emerging pollutants have been detected in aquatic ecosystems, mostly in the ng/L or µg/L range [1,2,3,4,5]. Although present at low concentrations, different environmental monitoring studies have already reported Pharmaceuticals and Personal Care Products (PPCPs) in some ecosystems at levels that can potentially lead to negative impacts on aquatic organisms and disrupt the sustainability of the ecosystems [6,7,8]. Their occurrence in the environment raises concern not only considering human health but also on wildlife. Despite the increasing interest of this issue, there is still a serious lack of information on the environmental fate and impact of PPCPs in non-target species [9,10,11,12].

PPCPs enter the environment through different sources and pathways. Whereas Wastewater Treatments Plants (WWTPs) are able to efficiently decrease the load of certain PPCPs, they cannot ensure complete removal of several classes. Therefore, WWTPs effluents are important sources of PPCPs in the aquatic environment containing significant concentrations of this compounds and their metabolites [2,3,8]. Personal Care Products (PCPs) are ubiquitous compounds, being detected in aquatic environments more often and at higher concentrations than pharmaceuticals, [11]. However, there is still a paucity of data on PCP toxicity [11].

Although most PCPs have not been designed to be bioactive in vertebrates, several have been documented to act as Endocrine Disrupting Chemicals (EDCs) given their ability to act as exogenous signals and potentially disrupt hormone-controlled physiological processes of both vertebrates and invertebrates, even at low concentrations [13,14,15,16,17].

Despite the state of knowledge on the toxicity of several contaminants being adequate for risk assessment, most emerging compounds are still poorly characterized with respect to their fate, behavior, toxicity and impact in non-target organisms [3]. Hence, this study aimed at improving the knowledge on the adverse ecotoxicological effects of several PCPs. The selection of PCPs for the present study considered their importance, either by environmental levels, occurrence and reported effects or gaps on their ecotoxicological effects. Therefore, we selected representatives of different PCP groups: UV filter (4-Methylbenzylidene Camphor), disinfectants (Triclocarban) and preservatives (Propylparaben). The organic UV filter 4-methylbenzylidene Camphor (4-MBC) is frequently detected in WWTPs effluents, being widely used as a UV filter in sunscreens [18]. Due to the high lipophilicity (log K_ow_ = 3–7) and environmental stability, UV filters are known to bioaccumulate in fish at levels similar to Dichlorodiphenyltrichloroethane (DDT) and several Polychlorinated biphenyl compounds (PCBs) [19]. For these reasons, UV filters have been found in lipid tissue in fish at concentrations higher than 2 ppm, with bioaccumulation factors greater than 5000 (21 µg/kg in the whole organism at 0.004 µg/L of UV filter in water) [11]. Propylparaben (PP) is a preservative belonging to the parabens class and is frequently used in cosmetics [11]. In addition, PP is one of the most ubiquitous parabens in the environment [20,21]. On the other hand, triclocarban (TCC) is widely employed in toothpaste, soaps, skin creams, deodorants and plastics because of its antimicrobial properties [11]. However, it exhibits a significant environmental persistence, which is a reason for concern [11,22].

The overall aim of the present work was to contribute to the ecotoxicological risk assessment of three emergent pollutants, 4-MBC, propylparaben and triclocarban. Considering their high sensitivity and extensive validation as a high-throughput screening approach for testing priority pollutants’ toxicity, the embryonic development of the teleost fish zebrafish (*Danio rerio*) (Hamilton-Buchanan, 1822) and the equinoderm sea urchin (*Paracentrotus lividus*) (Lamark, 1816) were used as model bioassay organisms to address PCPs toxicity.

## 2. Results

**Figure 1:**
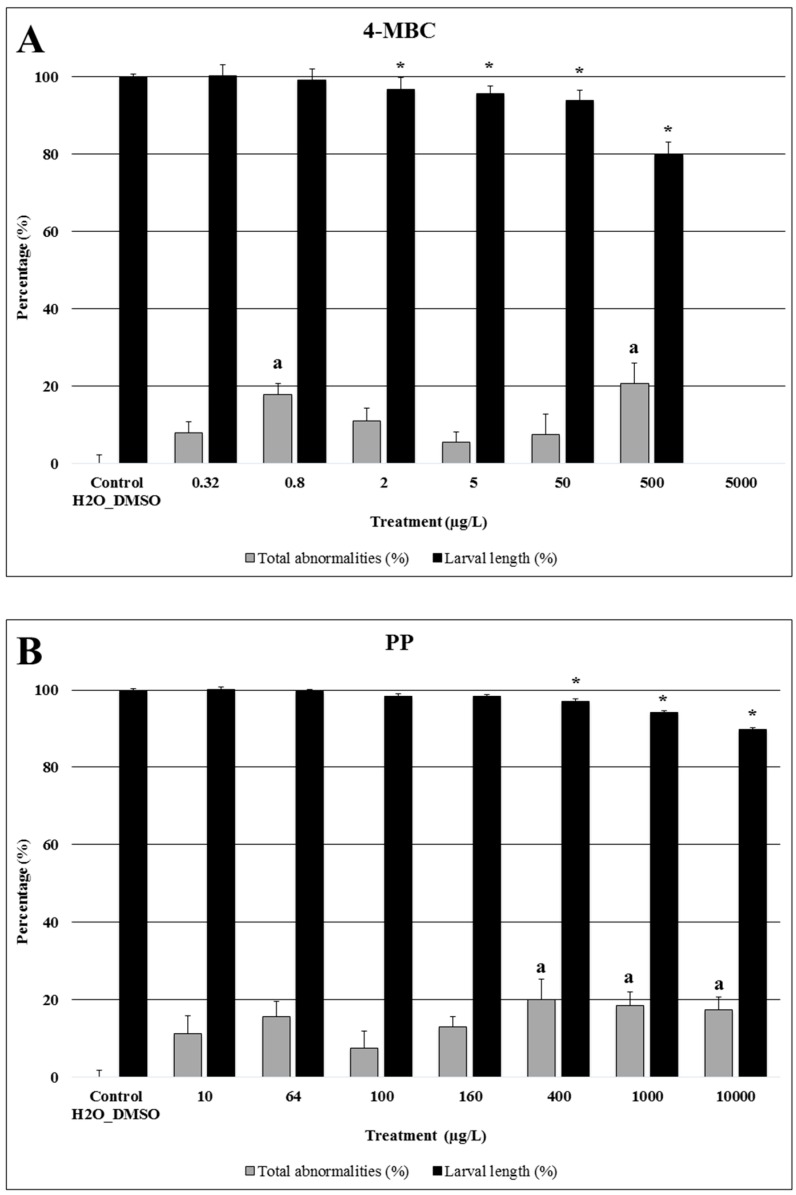

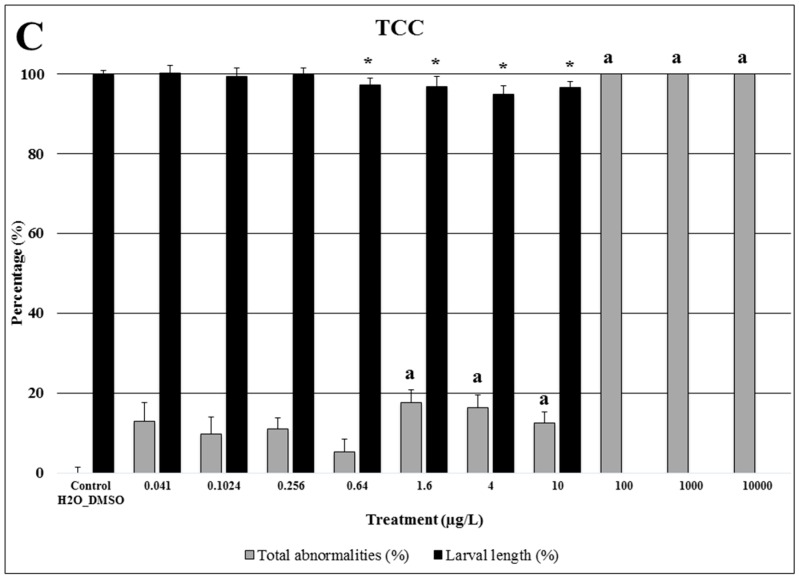
Larval length (%) and total abnormalities (%) of *Paracentrotus lividus* exposed to different concentrations of 4-Methylbenzylidene Camphor (4-MBC) (**A**), propylparaben (PP) (**B**), and triclocarban (TCC) (**C**) for 48 h. Controls and solvent controls were grouped. Results of first and second assays are normalized to the respective control assay, for both endpoints. Bars with the letter (a) or the symbol (*) indicate significant differences from controls (*p* < 0.05). The percentage of larval length inhibition data are expressed as mean ± SE (*n* = 480 for controls; *n* = 240 for 5 µg/L 4-MBC, 1000 µg/L PP, 10 µg/L TCC and 0.64 µg/L TCC; *n* = 120 for the other groups). The percentage of total abnormalities data are expressed as mean ± SE (*n* = 32 for controls; *n* = 16 for 5 µg/L 4-MBC, 1000 µg/L PP, 10 µg/L TCC and 0.64 µg/L TCC; *n* = 8 for the other groups).

The effects of exposure to 4-MBC, propylparaben and triclocarban on zebrafish embryo development at 8 and 80 hpf are summarized in Table 1, and on sea urchin larval length and morphological abnormalities in Figure 1.

### 2.1. 4-MBC

In the present study, no significant morphological abnormalities or pericardial edema were observed in zebrafish embryos until the end of the assay (80 hpf), despite a significant decrease in heart rate observed at the highest concentration (5 mg/L; *p* < 0.05), as well as a decrease in the hatching rate (Table 1). In addition, a significant increase of abnormal involuntary muscular contractions (*p* < 0.05) was observed at 5 and 0.5 mg/L as compared to the controls and other exposure groups.

Regarding sea urchin embryos exposed to the highest concentration of 4-MBC, all embryos died and therefore no measurements were performed at this exposure group (Figure 1A). Concentrations equal to or higher than 2 µg/L resulted in a significant decrease in larval length. A significant increase in the percentage of larvae with morphological abnormalities was observed at 0.8 and 500 µg/L, as compared to controls and other exposure groups (*p* < 0.05).

### 2.2. Propylparaben

Exposure to 10 mg/L of propylparaben proved to be lethal for all zebrafish embryos, while no significant increase of cumulative mortality rate (*p* > 0.05) was observed for the other treatments at the end of the assay (Table 1). Propylparaben concentrations equal to or higher than 8.5 mg/L induced a significant decrease in the percentage of zebrafish embryos at 75% of the epiboly stage at 8 hpf (*p* < 0.05). At 32 hpf, embryos exposed to 10 mg/L propylparaben showed an increase in the percentage of yolk-sac and tail abnormalities, as well as a decrease in heart rate, as compared with controls and other treatment groups. Exposure to 3.5 mg/L or higher increased the percentage of zebrafish embryos with tail abnormalities at 32 hpf. In addition, for the same range of concentrations, at 80 hpf, a significant (*p* < 0.05) increase in the percentage of zebrafish embryos with eyes, head, pericardial edema, tail abnormalities, yolk-sac and decrease of heart rate were observed. These effects might explain the high mortality of zebrafish embryos at the highest concentration tested and the development delay at the end of the assay. There was also a significant decrease of embryo hatching rate at 6 and 8.5 mg/L (*p* < 0.05), and, at 10 mg/L, all embryos died inside the chorion, at nearly the end of the assay.

As this compound was revealed to be less toxic than the other two selected PCPs, a new set of intermediate concentrations (2.5× dilutions) was tested in a second assay for sea urchin. Hence, an exposure to concentrations equal to or higher than 400 µg/L of propylparaben resulted in a significant increase in the percentage of abnormal larvae and a significant decrease of larvae length (Figure 1B).

### 2.3. Triclocarban

Triclocarban significantly increased the cumulative mortality rate of zebrafish embryos at 350 µg/L or above, between 32 and 80 hpf (*p* < 0.05), being lethal (100% mortality) at concentrations equal to or higher than 850 µg/L (Table 1). As this lethal effect occurred close to the end of the assay, it was possible to record the hatching rate, with no significant differences among groups (*p* > 0.05). No significant effects (*p* > 0.05) were observed for the other analyzed endpoints. There was a decrease in heart rate of embryos exposed to 350 µg/L, but, due to a high standard error, did not differ significantly from control.

Similar to 4-MBC, triclocarban also induced significant effects in sea urchin larvae at low concentrations (Figure 1C). Exposure of embryos to concentrations equal to or higher than 100 µg/L resulted in a significant delay in embryo development with no larvae reaching the four-arm stage. Hence, all embryos exposed to 1 and 10 mg/L of triclocarban displayed a gastrula stage at the end of the assay. At 100 µg/L, only embryos at the prism stage were observed. Triclocarban at 0.64 µg/L or higher significantly decreased larval length at the end of the assay and increased the percentage of larvae with morphological abnormalities at 1.6 µg/L or above.

## 3. Discussion

Many PCPs are detected in the environment at concentrations that might pose a risk for aquatic ecosystems [11] (Table 2). These reports emphasize the importance of performing more detailed studies given the lack of knowledge on the effects of several groups of PCPs in non-target organisms. Some of these PCPs are used in large quantities and several studies have reported environmental persistence, endocrine disruption activity and potential for bioaccumulation [11].

The exposure of zebrafish embryos to selected PCPs in this study resulted in effects that were compound and concentration-specific, i.e., development abnormalities, decrease of heartbeat rate, among others. In addition, sea urchin embryos showed interruption and delay of development, decrease in larval length and morphological abnormalities. These effects are likely to impact survival by affecting locomotion, behavior, and the ability to escape from predation.

### 3.1. 4-MBC: An Ultra Violet Filter

The use of UV filters has increased during the past decade due to public concern for the effects of UV radiation. The consequences of UV filters for aquatic organisms remain poorly studied, due to the limited and fragmentary ecotoxicological studies available, which do not contribute to a reliable risk assessment of these compounds on aquatic ecosystems. However, several reports point to UV filters’ estrogenic activity at a range from 10^−5^ to 10^−6^ of those of estradiol, and similar to other xenoestrogens [14]. In addition, binding studies reported that the 4-MBC can bind to both ERβ and ERα isoform [14].

A seasonal variation of UV filter concentration in aquatic ecosystems seems to occur. This is related to recreational activities such as bathing and swimming that leads to UV filters washing off from the skin [18,23,24,25]. Although its use as a UV sunscreen component is not allowed in the USA [26], 4-MBC is still frequently used in sunscreens despite evidence of endocrine disruption effects [27,28,29,30].

Although a few studies have reported the impact of 4-MBC in aquatic organisms, there is a lack of information about the effects in initial embryonic development stages [30]. In zebrafish embryos exposed to high 4-MBC levels, 0.5 and 5 mg/L, a significant increase in abnormal involuntary muscular contractions was observed. In addition, exposure to 4-MBC at concentrations equal to or higher than 5 mg/L, induced development delay and an abnormal development in embryos, affecting heartbeats and delaying the hatching time.

In a previous study performed by Li et al. with dechorinated zebrafish embryos [30], an increase in mortality was reported at high levels (between 3.82 and 6.36 mg/L). Here, 4-MBC at 2.54 mg/L also increased the percentage of embryos with axial curvature. In the present study, no significant differences in cumulative mortality rate were observed at the concentration range reported in the Li et al. study. This may be associated with differences in the genetic background of the fish stock or the experimental design. In the Li et al. study, zebrafish embryos were exposed to 4-MBC after dechorination [30]. This may explain the high mortality rate reported. This example also supports the hypothesis that the lower sensitivity of zebrafish embryos to some chemicals, in comparison to sea urchin embryos, may be associated with the protection provided by the chorion. In the former study, 4-MBC-treated embryos showed an impact in the swimming capacity and no response to tactile stimulations, which can be partly attributed to abnormal axial formation [30]. Based on that study, the altered axial curvature and shorter body observed are likely related to failure in the notochord differentiation process. Moreover, in that study it was also reported that 4-MBC inhibited acetylcholinesterase, which could lead to an accumulation of acetylcholine and inactivation of their receptors, resulting in defects in axonogenesis and muscle formation. Thus, in the present study, the increase in the percentage of zebrafish embryos with abnormal involuntary muscular contractions may be related to muscular dysfunctions from acetylcholinesterase inhibition. These effects in locomotion can hypothetically compromise survival.

In a recent study, using a similar test design (48 h exposure of *P. lividus* embryos to 4-MBC) an 50%-Effective Concentration (EC50) of 854 µg/L and NOEC and Lowest Observed Effect Concentration (LOEC) values of 300 and 600 µg/L for larval length were reported [26]. In our study, exposure of sea urchin embryos to 4-MBC resulted in a significant decrease of larval length at a much lower 4-MBC concentration (LOEC = 2 µg/L), which is in the range of environmental concentrations (Table 2).

Moreover, according to the results of both endpoints for 4-MBC exposure, the lower LOEC reported in our study (0.8 µg/L) for *P. lividus* shows a clear effect at concentrations lower than those already reported for this specie and at the same range of concentrations detected in coastal areas (Table 2). The reason for these contrasting results is unclear. Genetic or sensitivity differences between organisms may explain these results, which emphasizes the importance of performing more studies to understand the potential toxicological effects and environmental impacts, in order to achieve more reliable risk assessments of UV filters.

Using larvae length as the endpoint of the sea urchin bioassay has numerous advantages in terms of continuous response, observer-independence and feasibility of using software to perform the measurements. These advantages not only speed up test readings but also result in a more than two-fold increase in sensitivity when compared to the classical morphological endpoints [32]. Larval abnormalities require a higher sample size and there are observer-dependent endpoints that may result in subjective microscopial inspection when considering the presence and the degree of the different abnormalities [32]. However, according to our results in Ribeiro et al., the classical endpoints reveal a higher sensitivity for the pharmaceuticals propranolol, simvastatin and sertraline [33]. This may be related to the bioactivity of pharmaceuticals or their behavior and mode of in non-target organisms. Hence, we considered that for a robust and more informative toxicity assessment, both endpoints (larval abnormalities and larvae length) should be used in sea urchin embryo bioassays.

An increase in 4-MBC concentration in intertidal zones is expected during the summer due to the use of the greatest amount of sunscreens during recreational beach activities. On the other hand, bathing season corresponds to the spawning period of many organisms, including sea urchin. Some studies report 4-MBC environmental concentrations similar to those that impaired sea urchin embryo development in our study [23,25]. As sea urchin is present in both intertidal and subtidal zones, we can not rule out the hypothesis that actual concentrations of 4-MBC and/or other UV filters can affect embryonic development of wild populations, compromising the development of this species and perhaps as well as of other sensitive *taxa*, and of other organisms of the trophic web that depend on them, since *P. lividus* is a key stone organism in many rocky shore ecosystems.

### 3.2. Propylparaben: A Preservative

Propylparaben is widely used in cosmetic formulations and often detected in aquatic environments [20]. Despite this, only a few studies evaluated long-term effects of parabens in aquatic organisms [11]. Parabens with longer hydrocarbon chains can induce more adverse acute effects and are in general more persistent in the environment. In fact, whereas methyl- and ethylparaben are rapidly transformed, propyl- and butylparaben require more time to biodegrade [20].

Some parabens such as benzyl-, butyl- and propylparaben have been found to induce low-level estrogenic effects in aquatic organisms [11,21,34] Propylparaben was reported to increase vitellogenin (VTG) levels in plasma and upregulate the transcription of VTG genes in male medaka. Intraperitoneal injections of propylparaben in rainbow trout also induced estrogenic responses with induction of VTG in male fish [31]. Moreover, propylparaben can also influence sexual differentiation, as reported in Mikula et al. in a juvenile zebrafish 45-days bioassay, resulting in a significant increase of the female:male sex ratio [35].

In the present study, all zebrafish embryos exposed to concentrations equal to or higher than 3.5 mg/L displayed abnormalities at the end of the assay. In a recent study, González-Doncel et al. exposed *Oryzias latipes* (Temminck and Schlegel, 1846) embryos to several concentrations of propylparaben and examined physiological and anatomical abnormalities in embryos, eleutheroembryos (13 days post-fertilization—dpf) and larvae (42 dpf) [36]. No early or late toxic effects were observed at concentrations bellow 1 mg/L propylparaben. Exposure to 4 mg/L consistently induced significant deleterious effects during medaka embryonic, eleuteroembryonic and larval development. Exposure to concentrations equal to or higher than 0.4 mg/L resulted in a significant increase in the mortality rate during the larval stage. These results are comparable to those reported in the present study for zebrafish and sea urchin embryos, revealing a greater sensitivity of these species than *Leuciscus idus* (Linnaeus, 1758) (NOEC 48 h = 5 mg/L) [37], *Pimephales promelas* (Rafinesque, 1820) (LOEC 7 d growth and reproduction = 2.5 mg/L) [34], *Dapnhia magna* (Straus, 1820) (EC50 48 h immobilization = 15.4 mg/L) [38] and *Pseudokirchneriella subcapitata* (Hindák, 1990) (EC50 48 h growth = 15 mg/L) [38]. Actual environmental concentrations of propylparaben [20,31] (Table 2) are lower than the effective concentrations observed in this study, and, therefore, no effects are expected upon acute exposure of embryos of these species in the environment. However, possible adverse effects cannot be disregarded after long-term exposure to propylparaben due to its potential to bioaccumulate in organisms and act as an endocrine disrupting compound [39].

### 3.3. Triclocarban: Bactericide and Antifungal Agent

There are only a few studies reporting triclocarban toxicity in aquatic organisms. However, recent short- and long-term studies for aquatic invertebrates and fish indicate a slightly higher toxicity of triclocarban compared to triclosan, the most commonly detected and studied disinfectant [6,11]. Benthic invertebrates could be affected by triclocarban given that this compound has the potential to sorption to sediment [11]. In addition, triclocarban bioaccumulates in aquatic organisms, which can lead to biomagnification through the food chain [22,40]. Moreover, triclocarban exhibits significant persistence in the environment and has been frequently detected in WWTP effluents and surface water over the last years at concentrations higher than triclosan (TCS) and its methyl derivate methyl-triclosan [11,22].

Recent studies indicate that triclocarban can behave as an endocrine disrupting chemical, affecting the transcription of steroid sex hormones in human cell lines [41,42]. It was also shown that a diet containing a mixture of triclocarban and testosterone, fed to male castrated rats, resulted in synergetic effects and increase of gonad weight, in comparison with the control diets or single compound diets [41]. Triclocarban does not seem to act as a ligand of androgen receptor but shows amplification of androgen’s activity both in in vivo and in vitro studies, suggesting that the mode of action does not involve modulation at the receptor level [31]. Furthermore, triclocarban affected the transcription of genes associated with thyroid hormone signaling in frog and rat cells [40]. Exposure of freshwater mudsnails, *Potamopyrgus antipodarum* (Gray, 1843), to triclocarban promoted a significant increase in the number of embryos produced per female at concentrations above 0.2 µg/L. Moreover, a significant increase was also reported in the number of unshelled embryos at concentrations equal to or higher than 1.6 µg/L when compared to controls [42]. In a study performed by Schultz and Bartell, a decrease in aggression was reported in *P. promelas* adult males exposed to triclocarban (1.6 µg/L) or to mixtures (560 ng/L TCS + 179 ng/L TCC and 1.6 µg/L TCS + 450 ng/L TCC). This effect was observed up to four days after the end of exposure, and may lead to a decrease in defense ability and reproduction success [43]. The effects reported in previous studies were observed at concentrations that impacted sea urchin embryos development in the present study.

Zebrafish embryos were affected by triclocarban at concentrations above environmental relevance. However, the impact in sea urchin larval length was observed at environmentally relevant concentrations (LOEC = 0.64 µg/L). Taken together with the available data in the literature, it strongly indicates that triclocarban is likely to impact the most sensitive taxa at environmentally relevant concentrations.

## 4. Material and Methods

### 4.1. Chemicals

4-MBC, Propylparaben and Triclocarban were purchased from Sigma-Aldrich^®^ (Saint Louis, MO, USA), Potassium chloride (CAS 7447-40-7, 99.0%), Calcium chloride (CAS 10043-52-4, 93.0%), Magnesium chloride hexahydrate (CAS 7791-18-6, 99.9%), Magnesium sulfate (CAS 7487-88-9, 99.5%) were purchased from Sigma-Aldrich^®^. Sodium chloride (CAS 7647-14-5, 99.5%), Sodium bicarbonate (CAS 144-55-8) and dimethylsulfoxide (DMSO) were purchased from Merck (Keniworth, NJ, USA).

### 4.2. Species Selection

#### 4.2.1. Zebrafish

Zebrafish (*Danio rerio*) belongs to the Cyprinidae family, Actinopterygii class, and is a tropical cypriniforme fish, native to the rivers of India and south Asia [5]. This organism has been largely used as a model species for toxicological purposes due to its small size, short life-cycle, high sensitivity to a a range of chemicals, easy maintenance and reproduction in laboratory conditions as well as the availability of tools (full genome sequence, regulatory sequences and expression profile) [44,45]. Furthermore, eggs are translucent, which makes the follow up of embryo development and their manipulation easier [46,47].

#### 4.2.2. Sea Urchin

Sea urchin (*Paracentrotus lividus*) is an herbivorous echinoderm (Echinoidea class) present on rocky bottoms from intertidal and subtidal zones. Sea urchin shows a well-characterized embryonic development [48]. Thus, together with other factors like high sensitivity, easy fertilization and reduced development time, as well as their abundance, ecological and commercial relevance, sea urchin embryos provide useful experimental models to developmental toxicology research [13,31,48].

### 4.3. Fertilization and Embryo Collection

#### 4.3.1. Zebrafish

The stock of female and male adult zebrafish was kept in a 250 L aquarium with dechlorinated and aerated water in a recirculation system with both mechanical and biological filters, at a water temperature of 28 ± 1 °C and under a photoperiod of 14:10 h (light:dark) [47]. The fish were fed four times per day by an automatic feeder with a commercial fish diet Tetramin (Tetra, Melle, Germany) supplemented with live brine shrimp (*Artemia* spp.).

For spawning, a group of adult males and females (2:1) was housed, overnight, in a breeding tank, inside a 30 L aquarium and under the same water and photoperiod conditions as the stock. On the following day, ovulation and fertilization were stimulated by the beginning of the light period [47].

The experiments conducted in this work were performed at Biotério de Organismos Aquáticos (BOGA, CIIMAR) aquatic animal facilities, in accordance to the guidelines of the Directorate-General of the Veterinary of Portugal (decree law 113/2013), based on the European directive of animal welfare 2010/63/EU of the European Parliament and of the Council of 22 September 2010, and was approved by Ethical committee of Centro de Investigação Marinha e Ambiental (CIIMAR).

#### 4.3.2. Sea Urchin

Sea urchins were collected in a clean rocky shore in the north of Portugal, Granja, Vila Nova de Gaia (N 41°2’26.18’’, W −8°39’2.24’’) and transported to the laboratory in a portable icebox containing seawater. *P. lividus* gametes were obtained by dissection of a single pair of mature adults and assessed for their quality under a Nikon eclipse 50i microscope (Tokyo, Japan). Eggs were suspended in artificial sea water (90 mL) until a dense suspension was formed. Artificial seawater was prepared according to Zaroogian et al. using potassium chloride (0.67 g/L), calcium chloride (1.36 g/L), magnesium chloride hexahydrate (4.66 g/L), magnesium sulfate (2.04 g/L), sodium chloride (24.6 g/L) and sodium bicarbonate (0.39 g/L) [49]. A few microliters of undiluted sperm were added to the egg suspension, and the contents were gently stirred to allow fertilization. The number of eggs present and fertilization success, indicated by the presence of a fertilization membrane, were recorded under a microscope. Toxicity tests were performed when the fertilization rate was above 97%.

### 4.4. Experimental Design and Embryo Bioassays

#### 4.4.1. Experimental Solutions

The experimental design of our study for zebrafish embryo bioassay and the selected endpoints were based on ecotoxicity test guidelines of the Organization for Economic Cooperation and Development (OECD), FET 236, and the US Environmental Protection Agency (EPA) [50].

Experimental concentrations of selected PCPs were chosen based on data from the literature, with 10x dilutions, in order to cover a wide range of concentrations, including environmental relevant concentrations (Table 3). To refine the initial findings, a new set of toxicity assays with 2.5× dilutions (sea urchin) or intermediate concentrations (zebrafish) were carried out after a first screening.

For each chemical bioassay, initially six treatments conditions were set up: an experimental control, a solvent control (DMSO) and four experimental concentrations, for both species’ bioassay (eight replicates per treatment), placed in two equal-organized 24-well plates [33].

Stock solutions of each compound were prepared by dissolving 4-MBC, Propylparaben and Triclocarban in the organic dissolvent Dimethylsulfoxide (DMSO). The experimental solutions were obtained by diluting the stock solutions in artificial seawater (sea urchin assays) or in freshwater (zebrafish assays). All solutions were prepared in order to have a final DMSO concentration of 0.01%.

#### 4.4.2. Zebrafish

The static-water renewal toxicity test with zebrafish was performed according to OECD 236. For each replicate, 10 fertilized eggs were selected by magnifying glass observation and transferred to 24-well plates filled with 2 mL of freshly prepared solutions and controls per plate. The 24-well plates were incubated at 26.5 °C for 80 h and under the same photoperiod conditions as the zebrafish stock. Daily, the medium was renewed in order to keep oxygen and toxic nominal concentrations constant during the assay and to remove fungi or other organisms that could develop in the well.

Based on a set of characteristics present in zebrafish embryos at specific stages of development, three distinct periods were chosen to assess the effects of exposures: gastrula period (65%–75% epiboly stage), pharyngula period (prim 15–16) and larval stage (protruding-mouth), corresponding to 8, 32 and 80 h post fertilization (hpf), respectively [33,51]. Different parameters were recorded at each period of observation (Table 4).

Mortality was assessed by daily recordings during the entire exposure period and coagulated eggs or dead embryos were removed. Morphological abnormalities on head, tail, eyes or yolk-sac, pericardial edema, abnormal cell growth and 75% of the epiboly stage were rated as present or absent. In order to reduce the observation period, six embryos per replicate were randomly selected and assessed for the different morphological endpoints.

Heart rate was evaluated in one embryo per replicate using a stopwatch for 15 s, restarting the counting if the embryo moved. All zebrafish embryo/larvae observations were performed with a Leica EZ4 magnifying glass (Wetzlar, Germany).

#### 4.4.3. Sea Urchin

Fertilized eggs were placed in a 3 mL solution test in a concentration of 20 eggs/mL per well, within 30 minutes after fertilization. The 24-well plates were isolated with parafilm and embryos were incubated at 20 °C in the dark for 48 h. At the end of the exposure time, embryos were fixed by adding three drops of 37% formaldehyde and directly observed under an inverted microscope.

Although the percentage of abnormal larvae is the classical endpoint for sea urchin embryo bioassay [50], recent studies indicate that larvae length is an alternative and more sensitive endpoint [32,48]. Hence, two toxicity criteria were applied to record the embryogenesis success: larvae length and morphological abnormalities of randomly chosen individuals. Based on the results of Saco-Álvarez et al., the maximum larvae length was measured in 15 individuals per well in the *pluteus* stage (120 individuals per treatment) and morphological abnormalities were analyzed in 20 individuals per well (160 individuals per treatment) [32].

**Figure 2:**
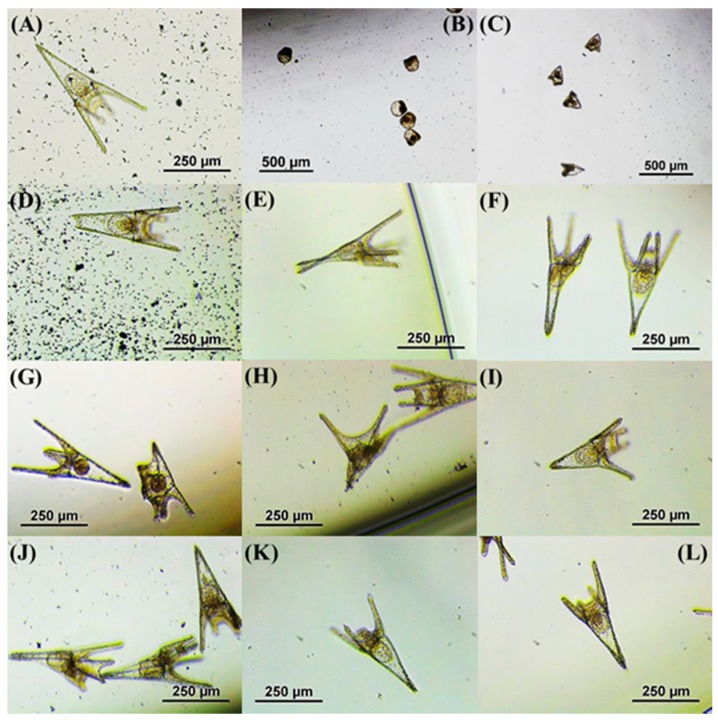
Embryonic stages and developmental abnormalities of the sea urchin *Paracentrotus lividus* observed in this study after 48 h of incubation under controlled conditions of temperature and light: (**A**) normal larva at pluteus stage; (**B**) gastrula stage; (**C**) prism larvae; (**D**) separated tip; (**E**,**F**) crossed tip; (**F**,**G**) fused arms; (**G**) general abnormal larvae; (**H**,**I**) abnormal arms orientation; (**J**) deformed arms; (**K**) arms absence; and (**K**,**L**) asymmetric arms.

Maximum larval length is defined as the distance between the apex and the end of the post-oral arm. Larvae were considered normal by the pyramid shape and four fully separated arms [32]. Abnormal larvae were counted separately as undeveloped organisms or malformed larvae. Embryos in which development suffers a delay or a blockage at early stages were considered undeveloped organisms. Changes in the normal sea urchin development at the end of the assay were recorded and presented as abnormal larvae; this included crossed tip, separated tip, folded tip, fused arms, deformed arms, abnormal arm orientation, absence or asymmetric arms (Figure 2).

*P. lividus* larvae observations were performed with a Nikon Eclipse 5100T inverted microscope equipped with a Nikon D5-Fi2 digital cam and a Nikon’s Digital Sight DS-U3 microscope camera controller (Tokyo, Japan). Larvae length was measured using NIS-Elements version 4.13 image acquisition software (Nikon, Tokyo, Japan).

### 4.5. Statistical Analysis

Data were analyzed using SPSS version 21.0 software (IBM Corporation, Armonk, NY, USA). All data were tested for homogeneity and normality using the Levene and Kolmogorov–Smirnov test, respectively. If these assumptions were met, differences between treatments were tested for significance by means of one-way factorial ANOVA followed by the Newman–Keuls multiple comparison test to compare the control groups and each of the exposed groups. If the homogeneity and normality were not met even after data transformation, the non-parametrical Kruskal–Wallis test, followed by the Games–Howell multiple comparison rank test, was used to test for significance. Data were presented as means ± standard error. The significance level was set at α < 0.05. According to the results of our experimental design, NOEC and LOEC of each compound were reported for both species (Table 2).

*D. rerio* statistical analysis was applied to the 80 hpf endpoints cumulative mortality, hatching rates, involuntary muscular contractions, pericardial edema and heart rate, abnormalities in head, eyes, yolk-sac and tail, as well as to the 8 hpf endpoints’ abnormal cell growth and 75% of the epiboly stage.

Control and solvent control were grouped for both species’ bioassays, when no significant differences between them were detected. The different abnormalities’ endpoints were also grouped as total abnormalities, in order to avoid non-significant results due to the assessment of these abnormalities’ sub-criteria separately. Therefore, we report total embryo abnormalities.

## 5. Conclusions

The present study shows that 4-MBC and triclocarban impact sea urchin development at environmentally relevant concentrations. Hence, we cannot disregard potential negative impacts of these compounds on the most sensitive taxa and/or developmental stages.

Taking into account the results of this study, sea urchin embryo bioassay reveled a higher sensitivity to the tested PCPs, in comparison with the zebrafish embryo bioassay. Larval length seems to be the most robust endpoint to evaluate the toxic response after exposure to various contaminants, but is not always the most sensitive. Additionally, results on both species contribute to further supporting the potential of early-life stages of aquatic species in high-throughput ecotoxicological tests and can be taken into account in risk assessment studies and hazard identification.

Given the findings of the present study, further research should be carried out in order to investigate chronic long-term effects of both 4-MBC and triclocarban.

## Figures and Tables

**Table 1:** Effects of 4-Methylbenzylidene Camphor (4-MBC), propylparaben (PP) and triclocarban (TCC) exposures in *Danio rerio.* Controls and solvent controls were grouped. Significantly different results from controls (*p* < 0.05) are marked with a symbol (*). Data are expressed as mean ± SE (*n* = 16 for 4-MBC controls; *n* = 32 for propylparaben and triclocarban controls; *n* = 8 for exposed groups).

Compound	Endpoints												
	Treatment (µg/L)	Mortalitiy Rate	Hatching Rate	Abnormal Cellular Growth	75%-Epiboly	Head Abnormalities	Eyes Abnormalities	Yolk-sac Abnormalities	Tail Abnormalities	Pericardial Edema	Heart Rate	Total Abnormalities	Abnormal Muscular Contractions
4-MBC	Controls	1.88 ± 1.0	95.0 ± 1.58	1.25 ± 0.85	90.0 ± 6.26	1.04 ± 1.04	1.04 ± 1.04	1.04 ± 1.04	2.08 ± 1.42	1.04 ± 1.04	126.75 ± 4.46	2.08 ± 1.42	0.0 ± 0.0
	5	7.5 ± 4.12	87.5 ± 3.66	7.5 ± 4.12	91.25 ± 3.98	4.17 ± 2.72	4.17 ± 2.72	4.17 ± 2.72	6.25 ± 3.05	2.08 ± 2.08	123.5 ± 3.58	6.25 ± 3.05	0.0 ± 0.0
	50	6.25 ± 2.63	92.5 ± 3.13	3.75 ± 2.63	95.0 ± 2.67	0.0 ± 0.0	0.0 ± 0.0	0.0 ± 0.0	6.25 ± 4.38	0.0 ± 0.0	120.0 ± 4.21	6.25 ± 4.38	0.0 ± 0.0
	500	5.0 ± 2.67	81.25 ± 4.79	3.75 ± 1.82	92.5 ± 3.13	4.17 ± 2.72	4.17 ± 2.72	4.17 ± 2.72	10.42 ± 3.05	2.08 ± 2.08	121.0 ± 3.53	10.42 ± 3.05	35.42 ± 11.55 *
	5000	2.5 ± 1.64	38.75 ± 7.66 *	1.25 ± 1.25	92.5 ± 4.91	4.17 ± 2.72	2.08 ± 2.08	2.08 ± 2.08	8.33 ± 4.45	2.08 ± 2.08	95.0 ± 3.0 *	10.42 ± 4.38	85.42 ± 3.78 *
PP	Controls	3.13 ± 0.95	92.19 ± 1.89	1.56 ± 0.65	97.81 ± 0.74	1.56 ± 0.87	1.56 ± 0.87	1.56 ± 0.87	3.13 ± 1.17	2.08 ± 0.99	143.0 ± 2.02	3.13 ± 1.17	–
	10	2.5 ± 1.64	87.5 ± 4.11	1.25 ± 1.25	96.25 ± 1.83	2.08 ± 2.08	2.08 ± 2.08	2.08 ± 2.08	2.08 ± 2.08	2.08 ± 2.08	144 ± 5.01	2.08 ± 2.08	–
	100	1.25 ± 1.25	90.0 ± 3.78	1.25 ± 1.25	98.75 ± 1.25	0.0 ± 0.0	0.0 ± 0.0	2.08 ± 2.08	0.0 ± 0.0	2.08 ± 2.08	146.0 ± 3.38	2.08 ± 2.08	–
	1000	4.37 ± 1.57	85.68 ± 5.91	1.25 ± 0.85	94.49 ± 1.76	3.13 ± 1.68	2.08 ± 1.42	2.08 ± 1.42	2.08 ± 1.42	2.08 ± 1.42	139.75 ± 3.36	4.17 ± 1.86	–
	3500	3.75 ± 1.83	66.25 ± 8.22	1.25 ± 1.25	96.25 ± 1.82	95.83 ± 2.73 *	89.58 ± 5.4 *	58.33 ± 7.04 *	91.67 ± 3.15 *	91.67 ± 4.45 *	124.5 ± 6.16 *	100 ± 0.0 *	–
	6000	1.25 ± 1.25	18.75 ± 3.98 *	1.25 ± 1.25	91.25 ± 3.98	100 ± 0.0 *	95.83 ± 4.17 *	83.33 ± 8.33 *	93.75 ± 6.25 *	100 ± 0.0 *	67.5 ± 7.98 *	100 ± 0.0 *	–
	8500	21.25 ± 7.89	2.5 ± 2.5 *	1.25 ± 1.25	87.5 ± 4.53 *	100 ± 0.0 *	100 ± 0.0 *	100 ± 0.0 *	100 ± 0.0 *	100 ± 0.0 *	70.29 ± 6.47 *	100 ± 0.0 *	–
	10,000	100 ± 0.0 *	0.0 ± 0.0 *	0.0 ± 0.0	87.5 ± 3.13 *	–	–	–	–	–	–	–	–
TCC	Controls	2.19 ± 0.87	96.88 ± 0.95	1.56 ± 0.79	97.81 ± 0.87	0.52 ± 0.52	0.52 ± 0.52	0.0 ± 0.0	0.0 ± 0.0	1.58 ± 0.88	130.5 ± 3.53	0.52 ± 0.52	–
	10	1.25 ± 1.25	98.75 ± 1.25	1.25 ± 1.25	98.75 ± 1.25	0.0 ± 0.0	0.0 ± 0.0	0.0 ± 0.0	0.0 ± 0.0	4.25 ± 2.78	118.5 ± 4.07	0.0 ± 0.0	–
	100	6.25 ± 1.8	91.11 ± 2.26	1.68 ± 1.01	93.61 ± 2.08	2.08 ± 1.42	2.08 ± 1.42	2.08 ± 1.42	3.12 ± 1.68	4.23 ± 1.89	128.0 ± 4.29	3.13 ± 1.68	–
	350	75.0 ± 12.81 *	91.25 ± 4.79	0.0 ± 0.0	97.5 ± 1.64	5.56 ± 5.56	0.0 ± 0.0	0.0 ± 0.0	33.33 ± 33.33	0.0 ± 0.0	72.0 ± 48.0	33.33 ± 33.33	–
	600	95.0 ± 5.0 *	92.5 ± 3.66	1.25 ± 1.25	97.5 ± 1.64	0.0 ± 0.0	0.0 ± 0.0	0.0 ± 0.0	16.67 ± 16.67	0.0 ± 0.0	–	16.67 ± 16.67	–
	850	100 ± 0.0 *	93.75 ± 2.63	1.25 ± 1.25	98.75 ± 1.25	–	–	–	–	–	–	–	–
	1000	100 ± 0.0 *	95.0 ± 2.76	0.0 ± 0.0	100 ± 00	–	–	–	–	–	–	–	–
	10,000	100 ± 0.0 *	83.75 ± 5.32	0.0 ± 0.0	98.75 ± 1.25	–	–	–	–	–	–	–	–

**Table 2:** Comparison of NOEC and LOEC values of selected PCPs reported in this study. Maximal concentrations of selected PCPs detected in surface water and in WWTPs influents and effluents (µg/L) retrieved from the literature.

Compound	Zebrafish		Sea Urchin		Maximal Concentration		
–	**NOEC**	**LOEC**	**NOEC**	**LOEC**	**WWTPs Influents**	**WWTPs Effluents**	**Surface Water**
4-MBC	50	500	0.32	0.8	6.5 [23]	2.7 [23]	* 0.799 [25]
PP	1000	3500	160	400	2.8 [20]	0.021[20]	0.207 [31]
TCC	100	350	0.256	0.64	50 [22]	>10 [11]	6.75 [11]

No Observed Effect Concentration (NOEC), Lowest Observed Effect Concentration (LOEC), Personal Care Products (PCPs), Wastewater Treatment Plans (WWTPs); * coastal areas.

**Table 3:** Tested concentrations in zebrafish and sea urchin exposures (µg/L).

Zebrafish			Sea Urchin		
4-MBC	PP	TCC	4-MBC	PP	TCC
5000	10,000	10,000	5000	10,000	10,000
500	8500	1000	500	1000	1000
50	6000	850	50	400	100
5	3500	600	5	160	10
–	1000	350	2	100	4
–	100	100	0.8	64	1.6
–	10	10	0.32	10	0.64
–	–	–	–	–	0.256
–	–	–	–	–	0.1024

**Table 4:** Endpoints recorded at 8, 32 and 80 hpf in *Danio rerio* bioassay.

Endpoint	8 hpf	32 hpf	80 hpf
Mortality rate	✓	✓	✓
75% of epiboly stage	✓	–	–
Abnormal cell growth	✓	–	–
Head abnormalities	–	✓	✓
Tail abnormalities	–	✓	✓
Eyes abnormalities	–	✓	✓
Yolk-sac abnormalities	–	✓	✓
Pericardial edema	–	✓	✓
Heart rate	–	✓	✓
Hatching rate	–	–	✓
^(1)^ Muscular involuntary contractions	–	–	✓

^(1)^ Only detected for 4-MBC exposure.

## Abbreviations

4-MBC	4-methylbenzylidene Camphor
DMSO	Dimethylsulfoxide
EC50	50%-Effective Concentration
EDCs	Endocrine Disrupting Chemicals
EE2	Ethynilestradiol
EPA	US Environmental Protection Agency
hpf	hours post fertilization
LOEC	Lowest Observed Effect Concentration
NOEC	No Observed Effect Concentration
OECD	Organization for Economic Cooperation and Development
PCPs	Personal Care Products
PP	Propylparaben
PPCPs	Pharmaceuticals and Personal Care Products
TCC	Triclocarban
TCS	Triclosan
VTG	Vitellogenin
WWTPs	Wastewater Treatment Plants
